# HIV, Hepatitis B and C viruses’ coinfection among patients in a Nigerian tertiary hospital

**Published:** 2012-08-08

**Authors:** Taiwo Modupe Balogun, Samuel Emmanuel, Emmanuel Folorunso Ojerinde

**Affiliations:** 1 Lagos State University Teaching Hospital

**Keywords:** HIV, Hepatitis B, Hepatitis C, coinfections, Nigeria

## Abstract

**Introduction:**

Hepatitis co-infection with HIV is associated with increased morbidity and mortality.

**Methods:**

This cross sectional study was carried out among HIV positive patients and HIV negative blood donors, HIV infected patients were recruited from the antiretroviral therapy clinics of the Lagos State University Teaching Hospital, in Nigeria. The diagnosis of HIV infection among patients and predonation screening of control blood donors was carried out using Determine1/2 screening rapid kits. (Inverness Medical, Japan). Reactive patients’ sera were confirmed with Enzyme Linked Immunosorbant Assay (Elisa) based immuuocomb1&11 comb firm kits (Orgenics, Israel). Hepatitis B surface antigen (HBsAg) and antibodies to hepatitis C virus (anti-HCV) were assayed using 4^th^ generation Dialab Elisa kits for patients and control sera.

**Results:**

Dual presence of HBsAg and anti-HCV was observed in 4(3.9%) of HIV infected patients, while 29(28.4%) and 15(14.7%) were repeatedly reactive for HBsAg and anti-HCV respectively. HIV negative blood donor controls have HBsAg and anti-HCV prevalence of (22) 6.0% and (3) 0.8% respectively. The prevalence of hepatitis co infection is higher among the male study patients 16(50%) than the female32 (45.7%).p > 0.001.Data analysis was done with statistical Package for social sciences (SPSS,9) and Chi square tests.

**Conclusion:**

This study reveals a higher risk and prevalence of HBV and HCV co infections among HIV infected patients compared to HIV negative blood donors p < 0.001.

## Introduction

Hepatitis B virus (HBV) and hepatitis C virus (HCV) infections cause chronic hepatitis, cirrhosis and hepatocellular carcinoma, all of which are of serious public health concern [1]. There is a heavy burden of HIV-HBV and HIV- HCV co infections in many regions of the developing world [2], Nigeria inclusive [3, 4]. Available data suggest 15-60% of the normal population in many African countries may be positive for one or more of the serological markers of hepatitis B virus [5]. The high prevalence of HBV infection in this region is thought to be due to horizontal transmission during childhood [5]. Individuals co infected with HIV and HBV are more likely to develop chronic hepatitis B and are at increased risk for liver related mortality [6]. Hepatitis C virus is the major cause of nonA, nonB hepatitis worldwide [7]. Hepatitis C co infection has been found to be more common in HIV+ve individuals and is associated with an increased mortality and renal morbidity [8]. In persons with HIV, HCV prevalence is estimated to be approximately 50% in the USA [9].

Recently, co infection between hepatitis C virus and HIV have been associated with rapid decline in the CD4 count, rapid progression of HIV infection and with increased morbidity and mortality [10]. Hepatitis co-infection with HIV accelerates disease progression in both HCV and HBV and also increases the risk of antiretroviral drug associated hepatotoxicity [11]. With an increase use and accessibility of highly active antiretroviral therapy among HIV positive patients in sub Saharan Africa, co-infection with these viruses could contribute significantly to continuing morbidity and mortality among this group of patients over the coming years.

The significant advancing in HIV management and survival have led to the recognition of chronic hepatitis as the pre eminent co morbid illness which now accounts for the majority of non AIDS related deaths in this population. To define the magnitude of this burden, we have examined the prevalence and risk of co infection with HBVand HCV among Nigerians with HIV infection. The result of this study would provide the baseline for future larger studies.

## Methods

After obtaining ethical approval from the Lagos State University Teaching Hospital (LASUTH) health research ethics committee, a cross-sectional study was conducted in March - August 2006. The study population was adult Nigerian HIV infected patients attending the antiretroviral therapy clinics. Informed consent was obtained from study subjects before specific structured questionnaires were administered to capture demographic data and risk factors which predispose to acquisition of both HBV and HCV co infections.

About 5millilitres of blood was collected from each patient and control subjects by venepuncture. The sera from the blood samples were separated and stored at −20°C until tested. Samples were brought to room temperature prior to testing and analyzed according to manufacturer's recommendations at the Blood Screening Centre of LASUTH.

Each serum was analyzed for the presence of HBsAg and anti-HCV using commercially available 4th generation Enzyme Limited Immuno Assay (ELISA) kits (Dialab, Austria) with 99.87% specificity and100%sensitivity. The diagnosis of HIV was made in patients using WHO approved Determine1/2 very rapid kits with100% sensitivity and 99.6% specificity. Positive serostatus was confirmed with ELISA based Immunocomb I &II comb firm kits (Orgenics, Israel) The data were analyzed using a statistical package for social sciences (Version 9, SPSS). Chi-square test was used to assess the significance of differences among groups. A value of less than or equal to 0.001 was considered significant in all statistical comparisons.

## Results

**Figure 1:**
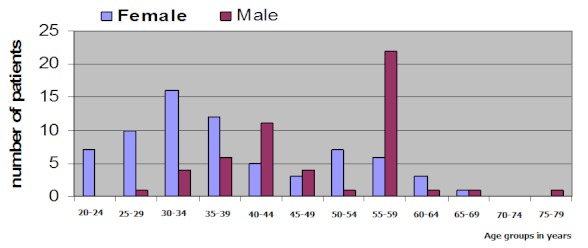
Age and sex distribution of patients

One hundred and two HIV infected patients comprising 32 (31.4%) males and 70 (68.6%) females with M: F ratio of 1:2.2 was enrolled for this study. Table 1 shows the dual presence of HBsAg and anti-HCV in 4(3.9%)study patients while 29 (28.4%) tested positive for HBsAg and 15 (14.7%) for anti HCV. The control subjects have a much lower prevalence of 22(6.0%) and 3(0.8%) for HBsAg and anti-HCV respectively while dual presence of HBsAg and ant-HCV was 0% prevalence. The difference in prevalence of hepatitis between the control and subjects is statistically significant p< 0.001 In Figure 1, there are more female patients in the younger age group with a peak at 30-34years while the males are older with a peak in the 55- 59 years age group. The age range of the patients is 20-79 with a mean of 38years. More male patients 16(50%) have hepatitis co infection than the female32 (45.7%). The difference is however not statistically significant p > 0.001 HIV/HBV co infected patients were more likely to be male12 (75%) while HIV/HCV co infected patients were more likely to be female12 (37.5%). In Table 2, triple infection with HIV/HBV/HCV was higher among the female 3 (9.4%) than the male patients 1 (6.3%).The difference is not statistically significant p > 0.001.

**Table 1:** Prevalence and Risk of HBsAg and Anti-HCV among HIV Patients and Controls

Hepatitis Status	Patients n = 102%		Controls n = 362%		X2	P- value
**HBsAg**					41.6	<0.001
Positive	29	28.4	22	6.0		
Negative	73	71.6	340	93.9		
**Anti-HCV**					40.5	<0.001
Positive	15	14.7	3	0.8		
Negative	87	85.2	359	99.2		
**HBsAg/antiHCV**					40.5	>0.001
Positive	4	3.9	-			
Negative	98	96.1	362	100		

**Table 2:** Gender Prevalence of HBsAg and Anti-HCV among study subjects

Gender	Hepatitis Negative n%	AntiHCV Positive n%	HBsAg Positive n%	AntiHCV &HBsAg +ve n%	Hepatitis Positive n%	Total Screened n%	Chi Square/p Value
Male	16 (50.0)	3 (18.8)	12 (75.0)	1 (6.2)	16 (50.0)	32 (31.4)	X2 = 1.81,P > 0.001
Female	38 (54.3)	12 (37.5)	17 (53.1)	3 (9.4)	32 (45.7)	70 (68.6)	
Total	54	15	29	4	48	102	

## Discussion

Our study examined the prevalence of HBsAg and anti-HCV among HIV infected patients and healthy HIV negative blood donors. We observed HBsAg and anti-HCV positivity of 29 (28.4%) and 15(14.7%) respectively among HIV infected patients. Among healthy HIV negative blood donor controls, we observed HBsAg and anti-HCV prevalence of 22(6.0%) and 3(0.8%) respectively. The high prevalence of HBsAg 29(28.4%) and anti-HCV 15 (14.7%) among HIV infected patients in this study is comparable with reports by Forbi et al in North Central Nigeria [4], South Africa cohort [8], Senegal [12] and France [13]. The prevalence of HBsAg 3(6.0%) among the control blood donors in this study is comparable with reported figures from previous studies in Lagos 6.9% [14] Ile-Ife7.3% [15], Nigeria. The observed HIV/HBV co infection prevalence(28.4%) in this study is comparable with previous reported high figures in different parts of Nigeria; (Keffi 20.6% [4], Jos28.7% [16], Ilorin30.4% [17], Kano70.5% [18]) and India 33.8% [19]. Some previous reports of HIV/HBV co infection from South Africa 6% [20], Nigeria (Maiduguri15% [21], Lagos 9.2% [22], Niger Delta9.7% [23] and Thailand 8.7% [24] are however lower than observed figures in this study. Varying sample size, test kit sensitivity and specificity may be responsible for the differences in prevalence figures in this group of patients.

The prevalence of HBV/HIV co infection was found to be higher among male study subjects12 (37.5%) than females 17(24.3%) in this study p = 0.001. The difference is not statistically significant. This finding is compatible with previous reports from Jos, North Central Nigeria [16] and India [19]. This observation may have been accounted for by the fact that men are more likely to have multiple sex partners and also practice unprotected sex in our polygamous setting. We also observed a significantly higher prevalence of HCV antibody among HIV infected patients as compared to HIV negative blood donors (14.7%vs0.8%respectively), p< 0.001. The difference is statistically significant. The reason in difference may be due to shared modes of transmission of both viruses in the study patients. This observed high prevalence (14.7%) of HCV/HIVcoinfection among study patients is comparable with reports of previous studies in Nigeria (Keffi11.1% [4] Abuja 8.2% [24]), South Africa 13.4% [8], France 17% [13], and San Francisco 11.7% [26]. We also found that the prevalence of HIV/HCV co infection was higher among the female patients 12 (37.5%) than the male 3 (18.8%), p > 0.001. The difference is however not statistically significant. This observation is at par with a previous study by Lesi et al [22], but at variance with other reports [4, 25] with findings of older males being more co- infected in Nigeria. This higher rate of HIV/HCV co infection among females may have been accounted for by the fact that women of all ages are more likely than men to become infected with HIV and HCV during unprotected vaginal intercourse. We have observed that 4(3.9%) of the study patients have triple co infection with HIV/HBV/HCV in this study. Previous prevalence reports of triple co infection in this group of study subjects vary from Keffi7.2% [4], Ibadan 1% [27] in Nigeria, Kenya 0.3% [28] and France 1.6% [13]. The results in this study show that the prevalence of HBV and HCV in the population of persons living with HIV in Nigeria is higher than found in the HIV negative population. In conclusion, there is therefore a higher risk of HBV and HCV co-infections among HIV infected patients compared to HIV negative blood donors in this study p < 0.001. This high co infection rates among the study subjects demonstrate a correlation between these viral infections which could influence evolution of HIV, HBV and HCV diseases.

## Conclusion

We recommend that HIV positive patients should be routinely screened for HBV&HCV markers before initiation of highly active antiretroviral therapy as this practice would guide correct choice of drug combination. This would in turn reduce morbidity and mortality from antiretroviral drug associated hepatotoxicity among these patients.
